# Parkin-mediated mitophagy as a potential therapeutic target for intervertebral disc degeneration

**DOI:** 10.1038/s41419-018-1024-9

**Published:** 2018-09-24

**Authors:** Zengjie Zhang, Tianzhen Xu, Jiaoxiang Chen, Zhenxuan Shao, Ke Wang, Yingchao Yan, Congcong Wu, Jialiang Lin, Haoli Wang, Weiyang Gao, Xiaolei Zhang, Xiangyang Wang

**Affiliations:** 1 Department of Orthopaedics, The Second Affiliated Hospital and Yuying Children’s Hospital of Wenzhou Medical University, West Xueyuan Road 109#, Wenzhou, 325027 Zhejiang Province China; 2 Zhejiang Provincial Key Laboratory of Orthopaedics, Wenzhou, Zhejiang Province China; 3 The Second School of Medicine, Wenzhou Medical University, Wenzhou, Zhejiang Province China; 4 The Third Affiliated Hospital and Ruian People’s Hospital of Wenzhou Medical University, Wansong Road 108#, Ruian, Zhejiang Province China; 5 Chinese Orthopaedic Regenerative Medicine Society, Ruian, China

## Abstract

Intervertebral disc degeneration (IDD) is a complicated pathological condition blamed for low back pain. Mitochondrion is of vital importance for cellular homeostasis, and mitochondrial dysfunction is considered to be one of the major causes of cellular damage. Mitophagy is a cellular process to eliminate impaired mitochondria and showed protective effects in various diseases; however, its role in IDD is still not clear. Here, we explore the role of Parkin-mediated mitophagy in IDD. In this study, we found that Parkin was upregulated in degenerative nucleus pulposus (NP) tissues in vivo as well as in TNF-α stimulated NP cells in vitro. Knockdown of Parkin by siRNA showed that Parkin is crucial for apoptosis and mitochondrion homeostasis in NP cells. Further study showed that upregulation of Parkin by salidroside may eliminate impaired mitochondria and promote the survival of NP cells through activation of mitophagy in vitro. In in vivo study, we found that salidroside could inhibit the apoptosis of NP cells and ameliorate the progression of IDD. These results suggested that Parkin is involved in the pathogenesis of IDD and may be a potential therapeutic target for IDD.

## Introduction

Low back pain (LBP) is a complicated disorder and a leading cause of disability worldwide^1,2^. Besides, LBP is a common symptom in populations and occurs in all age groups, from children to the elderly population^1^. Intervertebral disc degeneration (IDD) has been proved as one of the most important causes of LBP^3^. Mechanical stress, inflammation, and ageing have been recognized as leading causes of IDD^4,5^; however, the definite aetiology and pathophysiology of IDD is still not clear.

IDD is predominantly characterized by the imbalance of extracellular matrix synthesis and degradation, as well as increased apoptosis and senescence in nucleus pulposus (NP) cells^6–9^. As reported, proinflammatory cytokines, such as IL-1β, IL-1α, TNF-α, and IL-6 were increased in degenerative intervertebral disc. These cytokines, especially TNF-α, may promote extracellular matrix degradation, chemokine production, and changes in intervertebral disc cell phenotype^4^.

Mitochondria are an essential source of ATP for cellular function, but when damaged, mitochondria generate a plethora of stress signals, which lead to cellular dysfunction and eventually programmed cell death^10–12^. It is reported that proinflammatory cytokines may promote the accumulation of dysregulated mitochondria leading to a sustained production of ROS which terminal contribute to the oxidative stress and cell death^13–15^.

Autophagy is a degradation process to combat with cellular stress, impaired organelles, and unwanted proteins could be degraded by autophagy in cells^16^. Mitophagy is a selective degradation of mitochondria by autophagy, which may help mitochondria to maintain homeostasis during cellular stress^17,18^. More and more evidences shown that autophagy could functional protect NP cells against mitochondrial pathway induced apoptosis^19,20^. And recent studies showed that mitophagy may protect chondrocytes against apoptosis and decreased the production of ROS via removing dysfunctional mitochondria^13,21^. However, the role of mitophagy in IDD is not clear yet.

Three classical pathways have been identified to be involved in mitophagy, including PINK1/Parkin, NIX/BNIP3, and FUNDC1 pathways^22^. A study by Williams et al. identified CpG island methylation of the PARK2 gene (encode Parkin protein) promoter in IDD patients^23^, indicating the association of Parkin with IDD. Therefore, we focused our study on the Parkin-mediated mitophagy in IDD.

In this study, we evaluated the expression of Parkin in degenerative NP tissues in vivo as well as in TNF-α stimulated NP cells in vitro, and Parkin was found upregulated in both situations. We also found that TNF-α may induce mitochondrion impairment and apoptosis in NP cells, while knockdown of Parkin by siRNA may aggravate these above. Salidroside (Sal) is a phenylpropanoid glycoside extracted from Rhodiola. Previously, we reported that Sal may promote mitophagy in PC12 cell lines^24^. Here, we demonstrated that Sal may ameliorate mitochondrion impairment and apoptosis in NP cells via Parkin-mediated mitophagy activation. Also, the therapeutic effect of Sal-induced mitophagy activation was confirmed in rat IDD model in vivo. Our study revealed that Parkin is involved in the pathogenesis of IDD and may serve as a potential therapeutic target for IDD.

## Results

### Parkin expression was upregulated in degenerative NP tissues and TNF-α stimulated NP cells

**Fig. 1:**
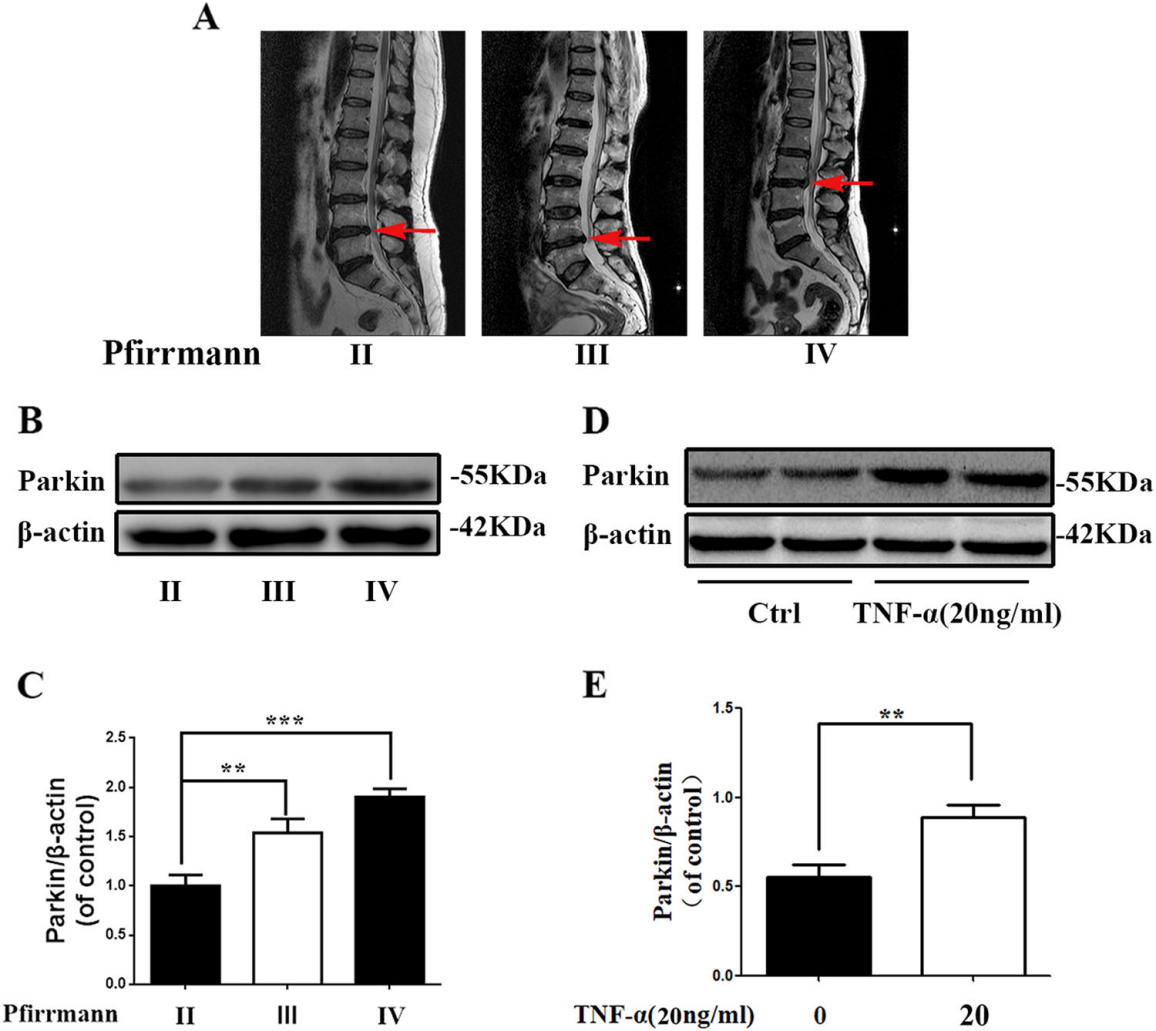
The expression of Parkin is increased in degenerated human NP cells and TNF-α treated rat NP cells. **a** Representative MRI images of three different degrees of IDD patients. **b** The expression of Parkin from NP cells of different degrees of IDD patients was analyzed by western blot. **c** Quantification of Parkin immunoblots. **d** The expression of Parkin from TNF-α treated NP cells was analyzed by western blot. **e** Quantification of Parkin immunoblots. Data represent mean ± SEM of three independent experiments, each done in triplicate. Significant differences between groups are indicated as ***p < 0.001, **p < 0.01, *p < 0.05

In order to investigate the relationship between IDD and Parkin, human NP tissues from different IDD degrees were collected and expression of Parkin in human NP tissues was evaluated by western blots. According to the results, as the degree of degeneration increases, the expression of Parkin was markedly increased in NP tissues (Fig. 1a–c). Meanwhile, TNF-α, a common elevated cytokine in degenerated disc tissues, was applied to establish IDD in vitro^4^. The results showed that the expression of Parkin in TNF-a stimulated NP cells is higher than that in un-stimulated NP cells (Fig. 1d, e).

### TNF-α stimulates mitochondrial ROS generation, apoptosis, and autophagy in NP cells

**Fig. 2:**
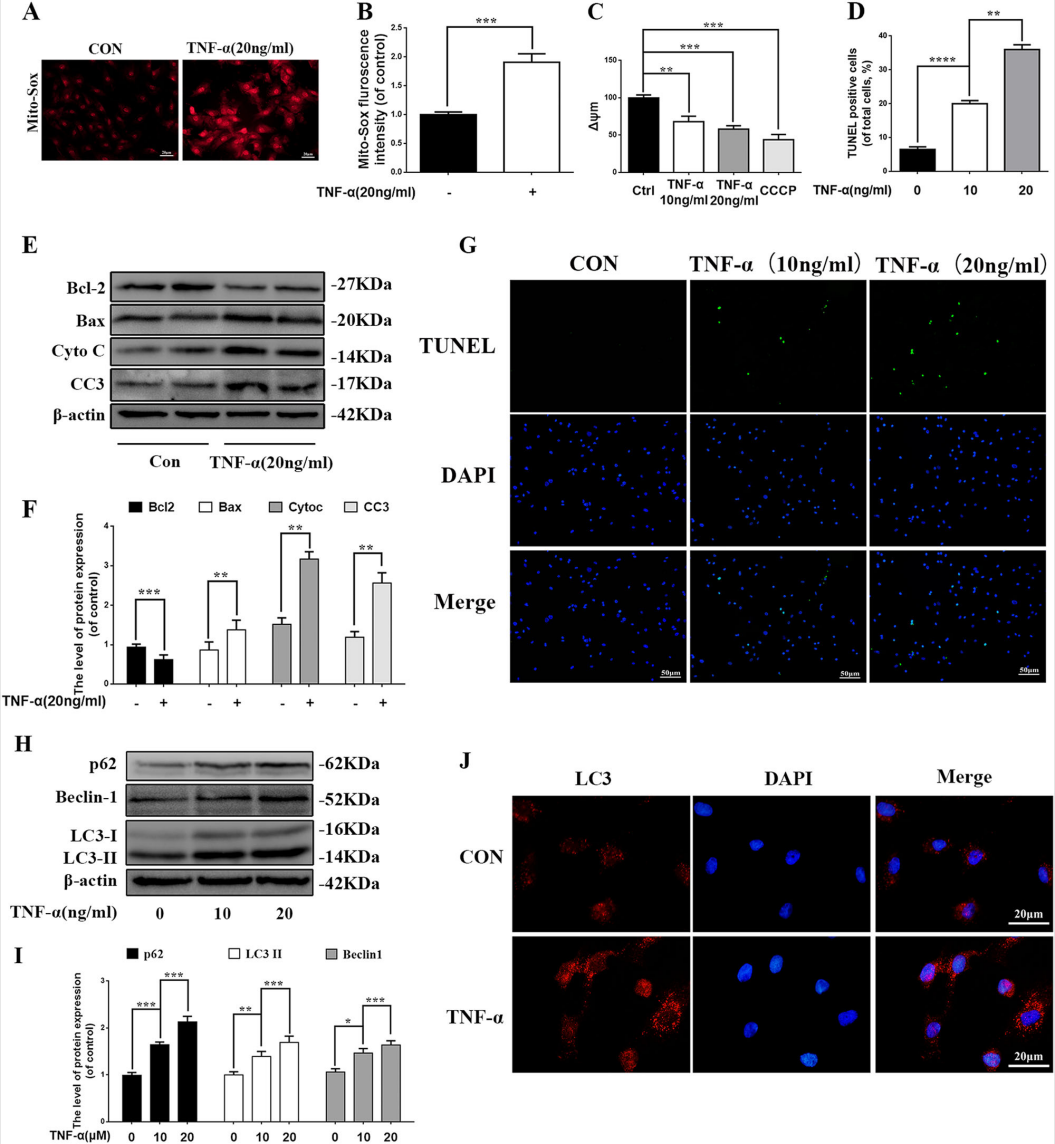
TNF-α treatment stimulates ROS generation, mitochondrial dysfunction, apoptosis, and autophagy in NP cells. NP cells stimulated with TNF-α for overnight. **a**, **b** TNF-α induced mitochondria ROS in NP cells were analyzed by Mito-Sox stains. And the fluorescence of Mito-Sox was quantified. **c** JC-1 was applied to measure the ΔѰM loss in TNF-α treated NP cells and analyzed by flow cytometer. CCCP was used as positive control. **d**, **g** TUNEL assay was performed in NP cells treated with different concentration of TNF-α (original magnification ×200, scale bar: 50 μm) and the quantification of apoptotic positive cells. **e** The expression of apoptosis related proteins such as Bcl-2, Bax, Cyto C, and Cleaved-caspase3 from TNF-α treated NP cells were analyzed by western blot. **f** Quantification of Bcl-2, Bax, Cyto C, and Cleaved-caspase3 immunoblots. **h** The expression of autophay related proteins such as p62, Beclin1and LC3 from TNF-α treated NP cells were analyzed by western blot. **i** Quantification of p62, Beclin1and LC3 immunoblots. **j** Immunofluorescence of LC3 in TNF-α treated NP cells were observed by fluorescence microscope (OLYMPUS). Data represent mean ± SEM of three independent experiments, each done in triplicate. Significant differences between groups are indicated as ***p < 0.001, **p < 0.01, *p < 0.05

Mitochondria ROS generation, apoptosis, and autophagy were evaluated in TNF-α stimulated NP cells. The results showed that mitochondria ROS was significantly increased in TNF-α stimulated NP cells (Fig. 2a), 20 ng/ml TNF-α resulted in significantly damage to mitochondria according to the changes of mitochondrial transmembrane potential in NP cells (Fig. 2c). The western blots results showed that cleaved-caspase3 and Bax (the pro-apoptotic index) was markedly increased while Bcl-2 (the anti-apoptotic index) was decreased in NP cells with TNF-α stimulation comparing to the untreated controls. We also found that Cytochorome C (Cyto C), a hemeprotein mainly presents in mitochondria and released into the cytoplasm when mitochondria are disrupted^25^, was significantly increased in cytoplasm of NP cells with TNF-α stimulation (Fig. 2e). TUNEL assay also confirmed that TNF-α promoted the apoptosis of NP cells (Fig. 2d, g).

Furthermore, western blot and fluorescence staining results showed that TNF-α not only promoted apoptosis (Fig. 2e, g), but also activated autophagy in NP cells (Fig. 2h, j). However, the autophagic flux was impaired since LC3 and beclin-1 were elevated while p62 was also elevated in TNF-α stimulated NP cells. In a conclusion, these results suggest that NP cells under TNF-α stimulation exhibits mitochondria impairment, increased ROS production, increased apoptosis, and activated autophagy.

### Knockdown of Parkin promotes apoptosis in TNF-α stimulated NP cells

**Fig. 3:**
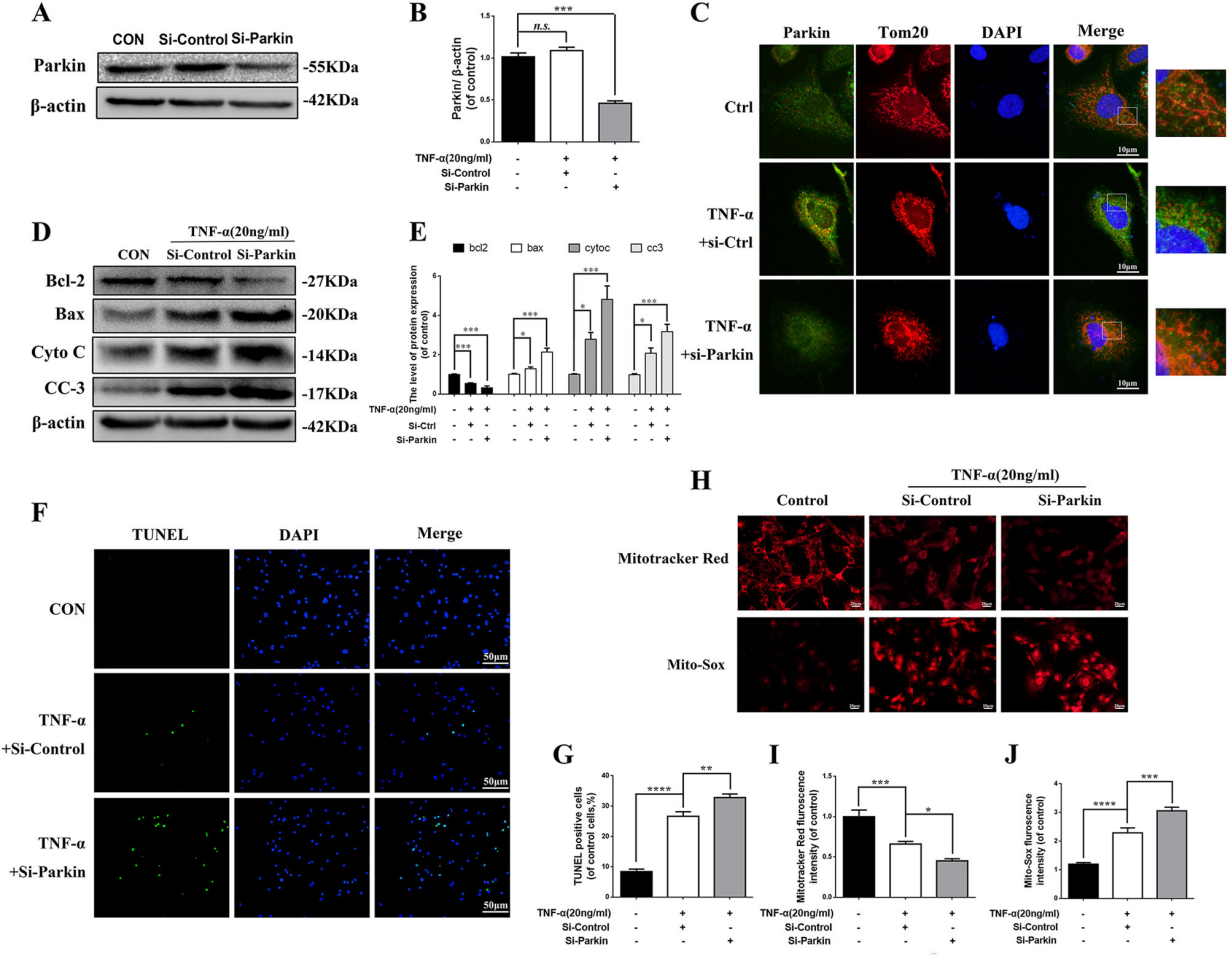
The expression of Parkin is tightly associated with mitochondrial dysfunction and apoptosis in TNF-α stimulated NP cells. NP cells were pretreated with Parkin target siRNA for 48 h followed TNF-α treatment overnight. **a** The expression of Parkin was evaluated by western blots. **b** Quantification of Parkin immunoblots. **c** Representative image of immunofluorescence double staining of Parkin and Tom20 in NP cells. **d** The expression of apoptotic proteins such as Bcl-2, Bax, Cyto C, and Cleaved-caspase3 was evaluated by western blots. **e** Quantification of Bcl-2, Bax, Cyto C, and Cleaved-caspase3 immunoblots. **f**, **g** TUNEL assay was performed in NP cells (original magnification ×200, scale bar: 50 μm) and the quantification of apoptotic positive cells. **h** The Mito-tracker Red probe was used to measure the mitochondrial membrane potential (MMP) and Mito-Sox was used to measure the mitochondria ROS in NP cells. **i** The fluorescence intensity of Mito-tracker Red in NP cells was quantified. **j** The fluorescence intensity of Mito-Sox in NP cells was quantified. Data represent mean ± SEM of three independent experiments, each done in triplicate. Significant differences between groups are indicated as ***p < 0.001, **p < 0.01, *p < 0.05

Since Parkin was elevated in TNF-α stimulated NP cells and degenerated NP tissues, we want to confirm whether increased expression of Parkin was correlated with increased apoptosis and ROS in TNF-α stimulated NP cells. We suppressed the expression of Parkin by siRNA. As shown in Fig. 3a, b, the expression of Parkin was successful inhibited. Double staining of Parkin and Tom20 (mitochondrial membrane protein marker) showed that siRNA reduced aggregation of Parkin in mitochondria in TNF-α stimulated NP cells (Fig. 3c). We also found that apoptosis was increased in TNF-α stimulated NP cells when the Parkin was knocked-down by siRNA according to western blots and TUNEL assay (Fig. 3d–g). Mito-Tracker red fluorescence showed significant decreased intensity in TNF-α group compared to control group, and siRNA can further reduce fluorescence intensity (Fig. 3h, i), suggesting that knockdown of Parkin may aggravate mitochondriol damage induced by TNF-α. We determined the mitochondria ROS generation in primary NP cells by Mito-SOX Red assay, the results showed that TNF-α treatment could increase the Mito-SOX fluorescence intensity and Si-Parkin could further increase the fluorescence intensity in NP cells under TNF-α stimulation (Fig. 3h, j). Together, these results suggest that Parkin may play a protective role in survival of NP cells by eliminating dysfunction mitochondria under TNF-α stimulation.

### Salidroside promotes Parkin expression, activates mitophagy, and suppresses apoptosis in TNF-α stimulated NP cells

**Fig. 4:**
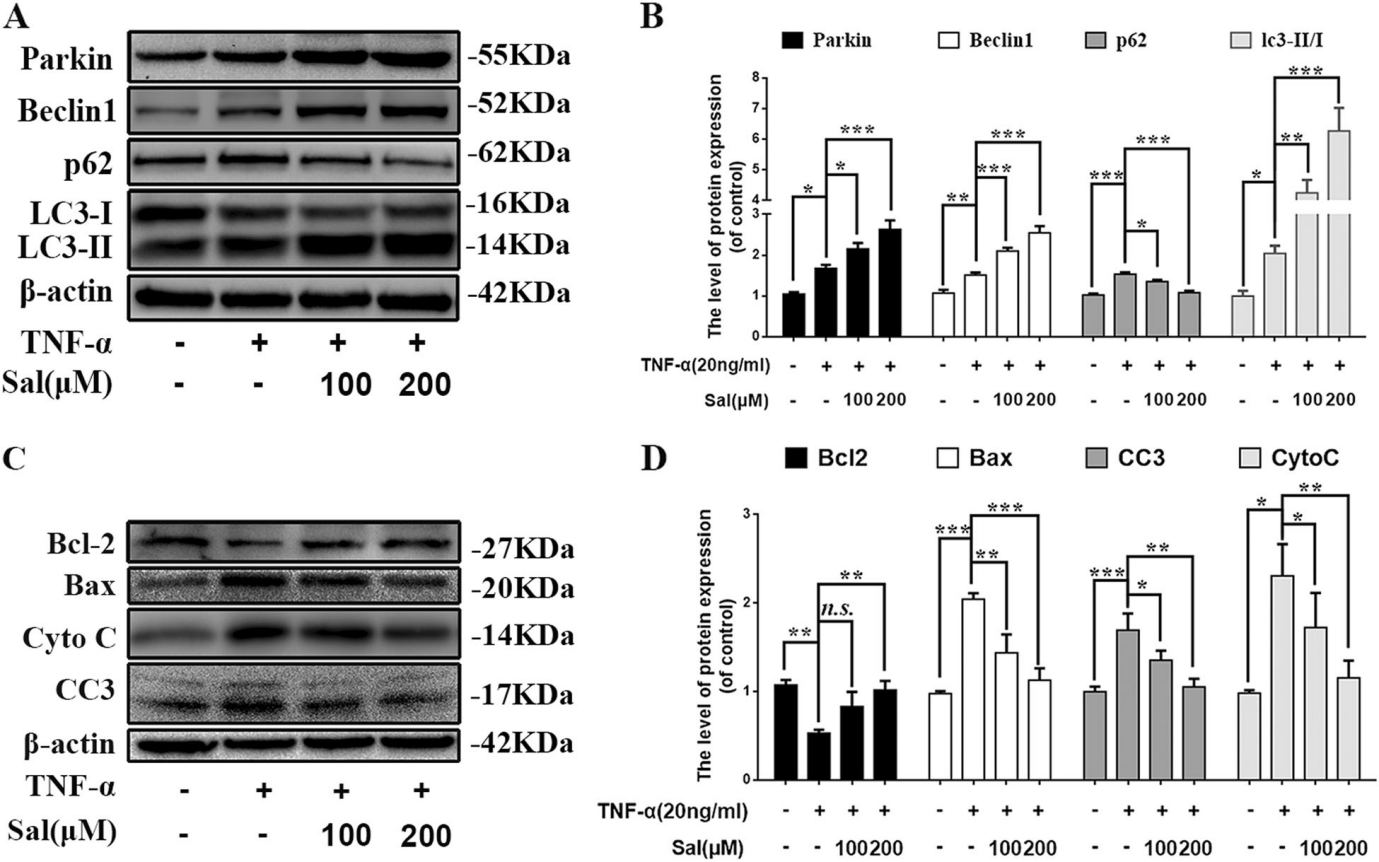
Salidroside treatment protects against TNF-α-induced apoptosis in NP cells. NP cells were treated with salidroside and TNF-α treatment overnight. **a** The expression of Parkin, Beclin1, p62, and LC3 in NP cells. **b** Quantification of Parkin, Beclin1, p62, and LC3 immunoblots in NP cells. **c** The expression of apoptotic proteins, such as Bcl-2, Bax, Cyto C, and Cleaved-caspase3 was evaluated by western blots. **d** Quantification of Bcl-2, Bax, Cyto C, and Cleaved-caspase3 immunoblots. Data represent mean ± SEM of three independent experiments, each done in triplicate. Significant differences between groups are indicated as ***p < 0.001, **p < 0.01, *p < 0.05

Previously, we found salidroside can regulate microglia polarization through autophagy in spinal cord injury^24^. Therefore, we want to investigate whether salidroside can eliminate damaged mitochondria and increase the survival of NP cells through Parkin-induced mitophagy in NP cells. We treated NP cells with different concentration of salidroside in the presence of TNF-α, the results showed that salidroside may promote Parkin expression in a dose dependent manner (Fig. 4a, b). Also, autophagy was activated as indicated by increased Beclin1 and LC3II expression; meanwhile, P62 expression was decreased in salidroside treatment group, suggesting autophagy flux was restored by salidroside treatment. The apoptosis in salidroside treated NP cells was assessed by Bcl-2, Bax, Cytochorome C (Cyto C), and Cleaved-caspase3 (CC3) western blot. We found that pro-apoptotic proteins (Cleaved-caspase3 and Bax) was significantly reduced and anti-apoptotic proteins (Bcl-2) was markedly increased in salidroside treated group in a dose dependent manner (Fig. 4c, d); while mitochondria related protein cytochromes C was reduced with salidroside treatment. Thus, these results reveal that salidroside may promote Parkin expression, activate autophagy and suppress apoptosis in TNF-α stimulated NP cells.

### Salidroside promotes mitophagy through Parkin

**Fig. 5:**
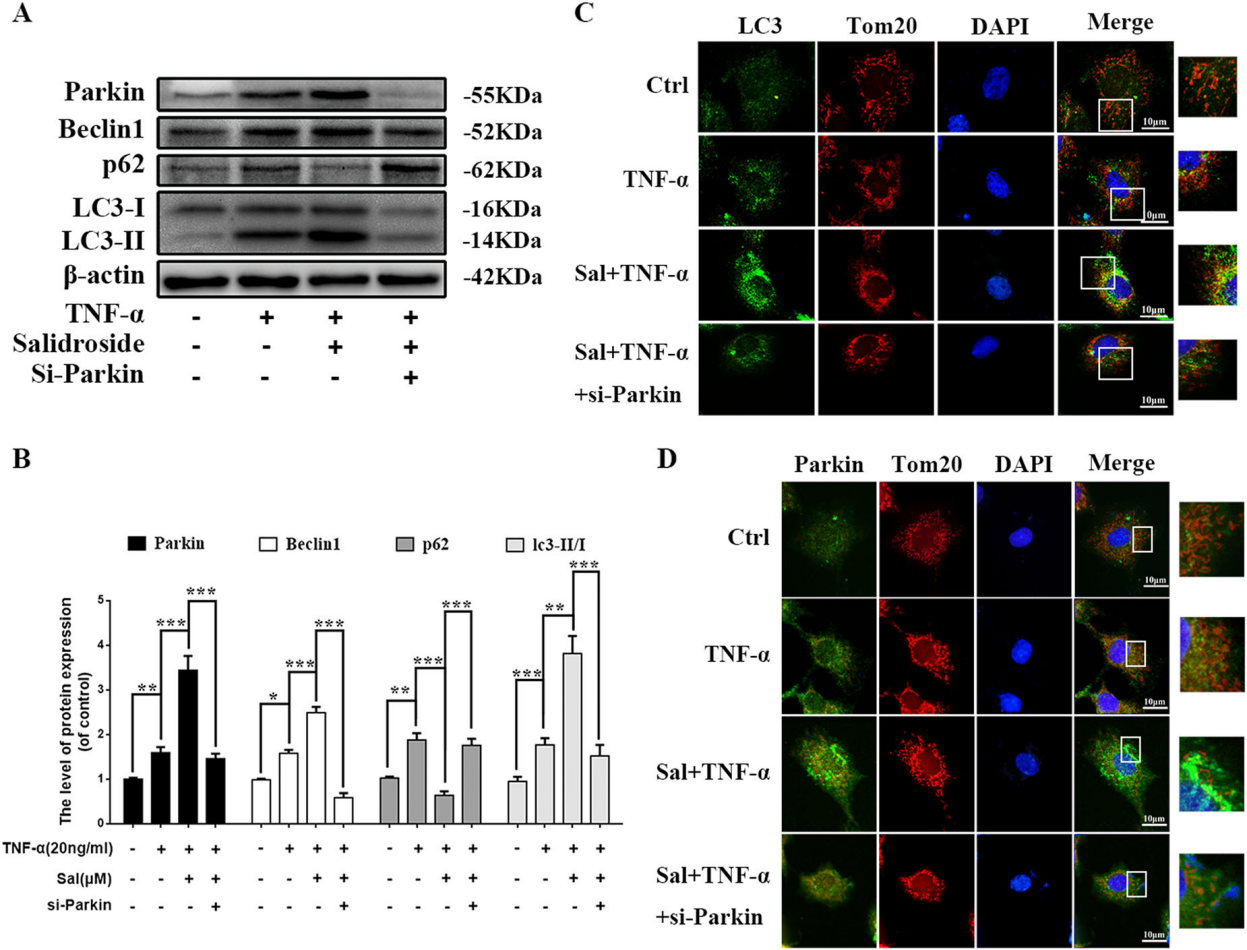
Salidroside regulates mitophagy by targeting Parkin expression in TNF-α stimulated NP cells. NP cells were pretreated with Si-Parkin for 48 h followed TNF-α treatment and salidroside treatment overnight. **a** The expression of Parkin, Beclin1, p62, and LC3 in NP cells. **b** Quantification of Parkin, Beclin1, p62, and LC3 immunoblots in NP cells. **c** Representative image of immunofluorescence double staining of LC3 and Tom20 in NP cells. **d** Representative image of immunofluorescence double staining of Parkin and Tom20 in NP cells. Data represent mean ± SEM of three independent experiments, each done in triplicate. Significant differences between groups are indicated as ***p < 0.001, **p < 0.01, *p < 0.05

The results above have shown that salidroside can upregulate expression of Parkin and promote autophagy in NP cells (Fig. 4), however it is not clear whether salidroside regulates mitophagy via Parkin, therefore Parkin was knockdown by siRNA to confirm the working mechanism of salidroside on mitophagy. As shown in Fig. 5a, when pretreated with Parkin siRNA, LC3-I, the essential component of autophagy initiation, was significantly decreased and the expression of the autophagic bubble formation indicator LC3II was also sharply decreased in NP cells. Beclin1 was also inhibited in NP cells when Parkin was suppressed. Meanwhile, the expression of p62 was significantly increased in si-Parkin group, suggesting that p62 was accumulated and autophagic flux was blocked. Mitophagy mediated clearance of depolarized/dysfunctional mitochondria in TNF-α stimulated NP cells could be determined by co-localization of autophagosomes. Autophagosomes were stained with LC3 and mitochondria were stained with mitochondrial outer membrane marker Tom20. The results showed that increased formation of LC3 positive autophagosomes (green) were co-localized with the mitochondria (Red) in salidroside treated group (Fig. 5c). However, when Parkin was knockdown by siRNA, co-localization of LC3 on mitochondria was rapidly decreased (Fig. 5c). The critical event in mitophagy is the translocation of cytosolic Parkin to the surface of defective mitochondria^26,27^. Consistent with this, we found that Parkin was accumulated on damaged mitochondria in TNF-α stimulated NP cells, and the accumulation of parkin was significantly increased in salidroside treated NP cells (Fig. 5d). After Parkin was inhibited, the accumulation of Parkin on mitochondria was decreased rapidly in TNF-α stimulated NP cells (Fig. 5d).

In a conclusion, Parkin is a crucial component in salidroside activated mitophagy which mediated clearance of damaged mitochondria in NP cells.

### Salidroside attenuates ROS generation and suppresses apoptosis through Parkin

**Fig. 6:**
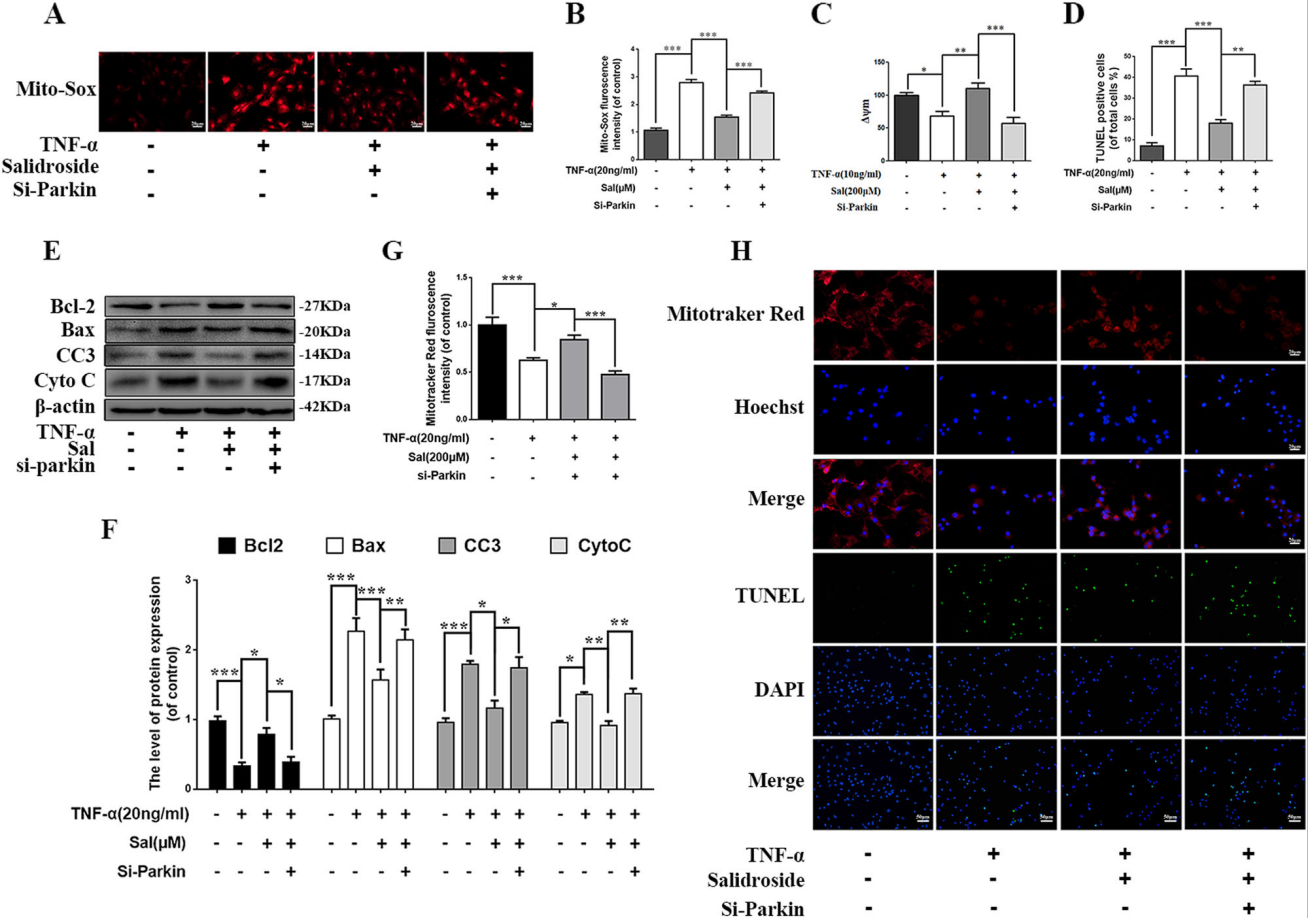
Salidroside inhibits ROS generation, mitochondrial dysfunction, and apoptosis through Parkin. NP cells were pretreated with Si-Parkin for 48 h followed TNF-α treatment and salidroside treatment overnight. **a** Mito-Sox probe was used to measure the mitochondria ROS in NP cells. **b** The Mito-Sox fluorescence intensity in NP cells was quantified. **c** JC-1 staining was applied to measure the ΔѰM loss in NP cells and quantified analyzed by flow cytometer (BD Accuri C6). **d**, **g**, **h** Mito-tracker Red probe was used to measure the mitochondrial membrane potential (MMP) and TUNEL assay was used to evaluated the apoptosis in NP cells and apoptotic positive cells was quantified. **e** The expression of apoptotic proteins, such as Bcl-2, Bax, Cyto C, and Cleaved-caspase3 was evaluated by western blots. **f** Quantification of Bcl-2, Bax, Cyto C, and Cleaved-caspase3 immunoblots. Data represent mean ± SEM of three independent experiments, each done in triplicate. Significant differences between groups are indicated as ***p < 0.001, **p < 0.01, *p < 0.05

To confirm the protective effects of salidroside in NP cells was via Parkin, NP cells were transfected with siRNA to suppress Parkin protein. The mitochondrial ROS was determined by Mito-Sox assay. As shown in Fig. 6a, fluorescence intensity of Mito-Sox was decreased in salidroside group compared to TNF-α group, whereas deletion of Parkin prevented the effect of salidroside in NP cells. Mitochondrial membrane potential was measured by JC-1 probe. There was higher mitochondrial membrane potential in salidroside group under TNF-α stimulation, nevertheless the mitochondrial membrane potential was decreased rapidly when Parkin was suppressed in NP cells (Fig. 6c). In addition, the mitochondrial homeostasis was evaluated by Mito-Tracker assay. Salidroside could increase the fluorescence intensity of Mito-Tracker Red, but the increment of fluorescence intensity was inhibited by siRNA of Parkin in NP cells (Fig. 6h, g). Therefore, these results suggest that salidroside maintains mitochondria homeostasis and inhibits ROS generation via Parkin.

To make sure whether salidroside alleviate TNF-α-induced apoptosis in NP cells by upregulating Parkin, the apoptosis was detected by western blot and TUNEL staining. We observed that anti-apoptotic related proteins (Bcl-2) was significantly decreased, and pro-apoptotic related proteins (Bax, Cyto c and cleaved-caspase3) were increased when Parkin siRNA was present in salidroside treated NP cells (Fig. 6e, f). And Parkin siRNA remarkedly reversed the decrease of apoptotic cells with salidroside treatment in TNF-α stimulated NP cells (Fig. 6d, h).

Take a conclusion, salidroside reduces the generation of ROS and inhibits the apoptosis of NP cells under TNF-α stimulation through Parkin-mediated mitophagy.

### Activation of Parkin by salidroside ameliorates intervertebral disc degeneration in vivo

**Fig. 7:**
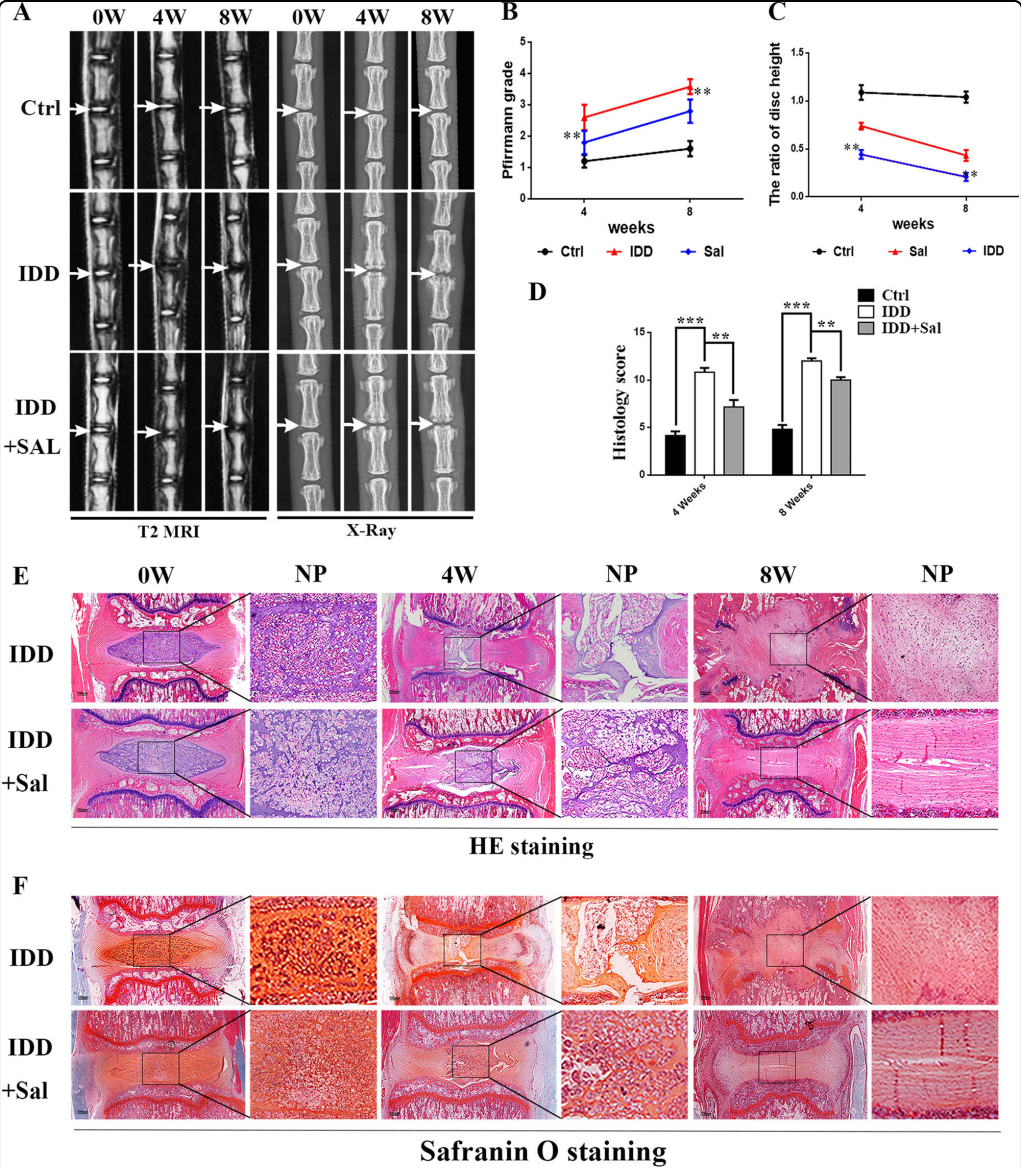
Salidroside ameliorates intervertebral disc degeneration in rRat punctured IDD model in vivo. Rat IDD model was established by stabbing the whole layer of annulus fibrosus (AF) of SD Rats through the tail skin using needles (26G) for 1 min. 0-, 4-, and 8-week degenerated discs were taken MRI, X-ray, and stained with H&E and Safranin O. **a** T2-weighted MRI and X-ray of a rat tail with a needle-punctured disc at 4 and 8 weeks post surgery (the white arrow: location of the needle-puncture disc). **b** The Pfirrmann MRI grade scores in three groups at week 4 and week 8. (Six rats at each time point for each group). **c** The disc height index (DHI) was determined in three groups at week 4 and week 8 (six rats at each time point for each group). **d** The histological grades evaluated at 4 weeks and 8 weeks post surgery in three groups (six rats per group). **e** Representative S-O staining of punctured disc in different group (original magnification ×40, scale bar: 100 μm). Three sections were randomly selected for quantification, with a representative example shown. **f** Representative HE staining of punctured disc in different group (original magnification ×40, scale bar: 100 μm). Three sections were randomly selected for quantification, with a representative example shown. Values of displayed are mean ± SEM of six rats per group. Significant differences between groups are indicated as ***p < 0.001, **p < 0.01, *p < 0.05

Based on the findings in in vitro study, we wanted to further verify the protective effect of activation of Parkin by salidroside on IDD in vivo. Puncture-induced IDD rat model was applied. X-ray images and MRIs were taken at 0, 4, and 8 weeks after disc puncture surgery and salidroside treatment. Magnetic resonance is the gold standard for detecting degeneration of intervertebral discs^28^, decreased magnetic resonance signal intensity in the intervertebral disc portion represents degeneration of the intervertebral disc. We observed a decrease of MRI signal intensity in punctured discs (the white arrow), while the signal intensity in punctured discs of salidroside group is higher than that in IDD group at 4 weeks and 8 weeks post surgery (Fig. 7a, b) (*p* < 0.05). In addition, the Pfirrmann MRI grade scores, which indicate the degree of disc degeneration, were significantly lower in the salidroside treated IDD rats than in saline treated IDD rats at 4 weeks (*p* < 0.05) and 8 weeks (*p* < 0.05). Disc height index (DHI) was used to measure the changes in the height of the intervertebral space; disc space height will gradually decline with the progression of disc degeneration. We observed that DHI of punctured discs of IDD group was significantly decreased comparing to control group at 4 weeks and 8 weeks after puncture surgery, while DHI in salidroside group was higher than that in saline group at 4 weeks and 8 weeks (Fig. 7a, c).

Hematoxylin and eosin staining and safranin O staining of punctured discs showed that NP cells were gradually lost in NP cavity of punctured discs and NP cells were replaced with fibrous cells. Numerous loss of proteoglycans and glycosaminoglycans of NP tissue could be observed in punctured discs (IDD group) at 4 weeks and 8 weeks according to the safranin O staining. And there was severe lamellar disorganization or fragmentation in punctured discs. The above changes observed in punctured discs were alleviated in salidroside treated IDD group comparing to saline treated IDD group at 4 weeks. And the structure of NP tissues in punctured discs was better preserved in salidroside group comparing to saline group not only at 4 weeks but also at 8 weeks (Fig. 7e). In addition, histological score of punctured discs was observed lower in salidroside IDD group than IDD group at 4 weeks or 8 weeks (Fig. 7d). In a conclusion, salidroside has therapeutic potential for ameliorating the progression of IDD.

**Fig. 8:**
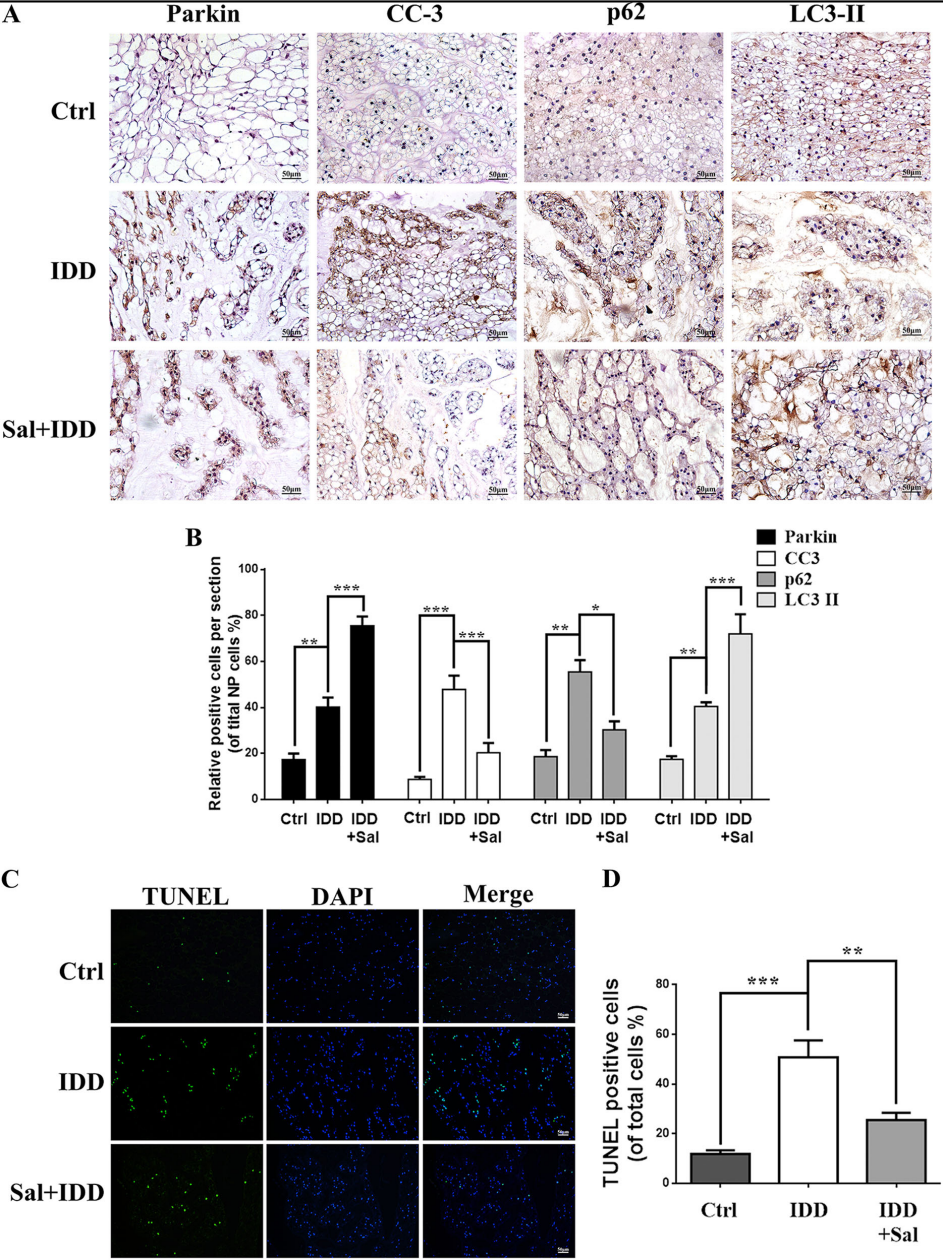
Salidroside ameliorates intervertebral disc degeneration through Parkin-mitophagy pathway in vivo. **a** The expression of Parkin, cleaved-caspase3, p62, and LC3 were evaluated by immunohistochemistry staining in punctured discs in different group. Representative images were selected and shown above. **b** Relative positive cells of Parkin, cleaved-caspase3, p62, and LC3 were quantified by image pro plus. **c** TUNEL assay was used to evaluate the apoptosis in NP cells of punctured discs in different group. **d** Apoptotic positive cells were quantified. Values of displayed are mean ± SEM of six rats per group. Significant differences between groups are indicated as ***p < 0.001, **p < 0.01, *p < 0.05

The apoptosis of NP cells in punctured discs was estimated by TUNEL assay and Cleaved-caspase3 immunohistochemical staining (Fig. 8a, c). We observed that apoptosis of NP cells was significantly increased in IDD group comparing to control group at 2 weeks post surgery. But upregulation of Parkin by salidroside reversed this pathological phenomenon in vivo (Fig. 8c, d). In order to investigate the Parkin-mediated mitophagy in vivo, we detect the expression of Parkin, LC3II, and p62 by the immunohistochemical staining (Fig. 8a). The results showed that increased Parkin by salidroside promote the expression of LC3 and decreased the accumulation of p62 in NP cells. These results suggest that salidroside-induced upregulation of Parkin is involved in the therapeutics of IDD by mediating autophagy.

## Discussion

LBP is plaguing 80% of the world’s population and IDD was recognized as one of the major causes blame for LBP. It is widely accepted that apoptosis of NP cells contributes to the progression of IDD according to recent studies, including ours^29–32^. Inflammatory cytokines, especially TNF-α, were significantly elevated in NP tissues of the degenerated IVDs and accelerate the progression of IDD. However, the mechanism of inflammatory cytokines-induced apoptosis in NP cells is still unclear, and there is no effective therapy targeting apoptosis in NP cells. In the progress of IDD, NP cells exposed to the condition of prolonged oxidative stress induced by inflammatory cytokines^33^ and contribute to the apoptosis^34^. Mitochondria dysfunction is tightly associated with oxidative stress. Therefore, we sought to establish the role of Parkin in eliminating dysfunctional mitochondria and its impact on ROS level and NP cells survival under TNF-α stimulation in this study.

We found that Parkin was elevated not only in human degenerated NP tissues but also in TNF-α stimulated rat NP cells. (Fig. 1). This is a novel finding and has not been previously reported in the field of IDD; the expression of Parkin was dependent on the degree of disc degeneration, indicating that there was an association between Parkin and IDD progression. The results that TNF-α promoted generation of ROS in NP cells in this study was consistent with the phenomenon that the accumulation of ROS was elevated in degenerated disc in vivo^33^. Intracellular ROS production mainly originates from mitochondria and membrane NADPH oxidase (Nox) system, especially the mitochondria, and mitochondria-induced ROS is tightly related to the ageing related disease such as IDD^33,35,36^.

In this study, we found that expression of Parkin was increased in TNF-α stimulated NP cells, meanwhile ROS generation was increased, and autophagy as well as apoptosis was activated (Fig. 2). Knockdown of Parkin by siRNA could increase the generation of mitochondria-induced ROS in NP cells and aggravate apoptosis induced by TNF-α (Fig. 3), suggesting that Parkin may involve in the eliminating of damaged mitochondria in NP cells and play an important role in regulation of ROS production and NP cells survival. Accidentally, salidroside was showed to increase the expression of Parkin and activate the autophagy in NP cells (Fig. 4). In order to explore the protective role of Parkin and working mechanism of salidroside in TNF-α stimulated NP cells, we used salidroside and Parkin siRNA to regulate the expression of Parkin in NP cells (Figs. 5 and 6). Our data showed that upregulating the expression of Parkin could reduce the production of ROS and increase the survival of NP cells. Consistent with our results, studies have also shown that Parkin-mediated mitophagy may eliminate damaged mitochondria and decrease the level of ROS in fibroblasts, primary chondrocytes, and lens epithelial cells^13,37,38^. In vivo experiments also demonstrated that salidroside mediated upregulation of Parkin plays a protective role in IDD in rats (Fig. 7).

Parkin was shown to be protective in IDD process, however why Parkin was upregulated in degenerated NP tissues as well as in TNF-α stimulated NP cells? From our view, the expression of Parkin is a responsed upregulation for NP cells to combat pathological conditions. During the IDD progression, NP cells suffer various environmental stress, i.e., oxidative stress (increased ROS); increased ROS may induce recruitment of Parkin and trigger Pink1/Parkin dependent mitophagy^39,40^. When Parkin was blocked, the generation of ROS was significantly increased in NP cells under pathological condition^13^, which has also been proved in our study. These results suggest that there was a cross-reaction between ROS and Parkin. However, the mechanism of Parkin reversely mediate the ROS generation remains unclear. Taken together, ROS may promote the overexpression and recruitment of Parkin and Parkin reversely suppress the generation of ROS.

In recent studies, autophagy has been thought to be a protective mechanism under pathological conditions through degrading intracellular damaged proteins or dysfunctional organelles. According to studies from our group^30–32^ as well as other groups^41–43^, autophagy plays an important role in protecting NP cells against apoptosis under pathological situation in vitro and postponing IDD process in vivo. Various drugs have been reported to induce selective mitochondria autophagy called mitophagy to eliminate the dysfunctional mitochondria. It is intriguing to see that the level of apoptosis, autophagy, and expression of Parkin were all increased under pathological condition with TNF-α stimulation (Fig. 2), we also observed that expression of p62 was increased in TNF-α stimulated NP cells in our study (Fig. 2). The ubiquitin associated protein p62, which binds to LC3, is an indicator of autophagic flux^44^, destroy any process of autophagy will lead to the impairment of autophagy flux which will further affect normal cellular function. Webster’s study showed that depleted Parkin expression could increase the expression of p62 in cells^45^, indicating Parkin deficiency may lead to impairment of autophagic flux. And impaired autophagic flux has been reported to contribute to apoptosis in various cells, such as retinal neuronal cells, myocardial cell^46,47^. Furthermore, ROS was reported to disturb autophagic flux and promote apoptosis during DTT-induced ER/oxidative stress in HeLa cells^48^. In our study, we found that ROS was elevated in NP cells with TNF-α stimulation. Therefore, we conclude that TNF-α may block the autophagic flux via generating ROS in NP cells and further influence the effects of Parkin guided clearance of damaged mitochondria and finally contribute to apoptosis in NP cells.

Although there are some novel findings in our study, two major questions are remained. First, although, we found that TNF-α may induce excessive ROS generation and affect the expression and localization of Parkin in NP cells, whether these effects are sololy mediated by ROS remains unclear. Therefore, tert-butyl hydroperoxide (TBHP), which is commonly used as an exogenous inducer of oxidative stress, should be used to investigate the role of Parkin on NP cells under oxidative stress alone. Second, as salidroside is a Chinese patent medicine that has many pharmacological effects, the protective effect of salidroside on NP cells may not only through upregulating Parkin, but also through other signaling pathways. Therefore, in order to further investigate the protection of Parkin in the processes of IDD, signaling pathways such as mTORC1 should be included in future studies.

In a conclusion, our study reveals that Parkin expression is associated with the progression of IDD, it is also involved in the clearance of dysfunction mitochondria via mitophagy in degenerated NP cells. Salidroside could suppress the generation of ROS and inhibit the apoptosis in NP cells via Parkin-mediated mitophagy. Thus, Parkin may become a therapeutic target for IDD. Finally, due to the association between Parkin and IDD, a wide range of drug screening targeting Parkin may support clinical treatment of IDD.

## Materials and methods

### Ethics statement

All surgical interventions, treatments, and postoperative animal care procedures were performed in strict accordance with the Animal Care and Use Committee of Wenzhou Medical University.

### Human NP cells

Human NP cells of different degeneration grades were isolated from different degenerated IVDs respectively to compare the expression of Parkin by western blot. IVDs tissues were cut up into 1 mm^3^ and washed for three times with phosphate-buffered saline (PBS) before digestion with 0.25% type II collagenase for 4 h at 37 ℃. Then wash the cell with PBS for three times after centrifugation. The cell suspensions were cultured in Dulbecco’s modified Eagle’s medium (DMEM) with 10% foetal bovine serum (FBS; Gibco, Waltham, MA, USA) and 1% penicillin/streptomycin (Invitrogen). The culture medium was replaced twice every week.

### NP cell culture

NP cells were extracted from healthy NP of young Sprague-Dawley rats (20 males, 100–150 g) according to the previous describe^30^, plated and expanded for 3 weeks at 37 °C and 5% CO_2_ in Dulbecco’s modified Eagle’s medium (DMEM) containing 20% foetal bovine serum (FBS; Gibco, Waltham, MA, USA) and 1% penicillin/streptomycin (Invitrogen). The culture medium was replaced twice every week, except in the case of primary cells, which were allowed more time (6.7 ± 1.4 days) to adhere prior to the first change of medium. Cells from the second passage were used in subsequent experiments.

### Cell viability assay

Cell viability was assayed with the cell counting kit-8 (CCK-8; Dojindo Co, Kumamoto, Japan) according to the manufacturer’s protocol. As previous description^30^, first, second-passage NP cells were plated in 96-well plates (8000 cells/cm^2^) and cultured in DMEM with 10% FBS at 37 °C and 5% CO_2_ for 24 h. After treatment, the cells were washed with phosphate-buffered saline (PBS), and then 100 µl of DMEM containing 10 µl of CCK-8 solution was added to each well, and the plate was incubated for an additional 2–4 h. The absorbance of the wells was then measured at 450 nm using a micro-plate reader.

### siRNA transfections

Validated siRNAs for Parkin and control siRNA were obtained from RiboBio Co., Ltd (Guangzhou, China). For transfection, when reaching 50–60% confluence NP cells, NP cells were transfected with Parkin siRNA according to the manufacturer’s protocol.

### Western blot assay

Total protein of was isolated from cells using RIPA buffer with 1 mM phenylmethanesulfonylfluoride (PMSF), and protein concentration was measured using the BCA protein assay kit (Beyotime). Thirty micrograms of protein was separated by sodium dodecyl sulfate-polyacrylamide gel electrophoresis (SDS-PAGE) and transferred to a polyvinylidene difluoride membranes (Bio-Rad, USA). Following blocking with 5% non-fat milk, the membranes were incubated with primary antibodies against cleaved-caspase3 (1:1000), Bax (1:1000), Bcl-2 (1:1000), LC3 (1:1000), p62 (1:1000), Beclin1 (1:1000), Cytochrome C (1:1000), β-actin (1:1000), and Parkin (1:500) overnight at 4 °C, followed by incubation with the respective secondary antibodies. The bands were detected with electrochemiluminescence plus reagent (Invitrogen). Finally, the intensity of the protein bands was quantified with Image Lab 3.0 software (Bio-Rad).

### Immunohistochemistry

After deparaffinization, sections of sample for immunohistochemistry were incubated with 3% H_2_O_2_ for 10 min and washed by PBS for three times. Then the sections were incubated with 0.1% trypsin for 20 min and washed by PBS for three times. Sections were blocked with 1% (w/v) goat serum albumin for 1 h at 37 °C, followed by primary Parkin, LC3II, P62, Cleaved-caspase3 antibody (Abcam, USA, 1:200) incubation at 4 ℃ overnight. Negative control sections were incubated with non-specific IgG. Next, the sections were washed by PBS for three times and incubated with HRP-conjugated secondary antibodies for 1 h at 37 °C. At least three sections from each specimen were observed. The rate of positive cells each section was quantitated by observers who were blinded to the experimental groups.

### Immunofluorescence

Cells were grown on coverslips, and the treated cells were washed with PBS for three times, then fixed with 4% paraformaldehyde for 15 min, permeabilized with 0.5% (v/v) Triton X-100 for 20 min, and blocked with 1% (w/v) goat serum albumin for 1 h at 37 °C. The samples were then probed at 4 °C overnight with antibodies against Parkin, LC3II, Tom20, cleaved-caspase3, and incubated for 1 h with a secondary antibody. Finally, nuclei were stained for 5 min with 0.1 g/ml 4’,6-diamidino-2-phenylindole (DAPI), and samples were washed and imaged with a fluorescence microscope. Protein expression was quantified by integrated optical density (IOD) with the Image-Pro Plus image analysis system.

### TUNEL assay

The terminal deoxynucleotidyl transferase (TdT) dUTP nick-end labelling (TUNEL) assay is a technique for measuring apoptotic DNA fragmentations. As previously described^31^, after being fixed with a freshly prepared 4% paraformaldehyde for 1 h, NP cells were incubated with 3% H_2_O_2_ and 0.1% Triton X-100 for 10 min and washed three times with PBS between steps. According to the manufacturer’s instructions, the cells were stained with the In Situ Cell Death Detection Kit (F. Hoffmann-La Roche Ltd., Basel, Switzerland) and DAPI. Finally, three random fields were imaged per slide with a fluorescence microscope (Olympus Inc., Tokyo, Japan).

### Reactive oxygen species assay

Cells were plated in six-well plates in complete culture medium. After 16 h, the cells (90% confluence) were washed with PBS. Then, the cells were incubated with reactive oxygen species (ROS) assay mixture according to the protocol of a reactive oxygen species (ROS) assay kit. Finally, three random microscopic fields were imaged per slide with a fluorescence microscope (Olympus Inc., Tokyo, Japan).

### Measurement of mitochondrial ROS

Mitochondrial ROS was measured by using Mito-SOX Red dye (Invitrogen, M36008). NP cells were plated in six-well plates in complete culture medium, after treatment with salidroside or without under pathological condition(TNF-α), NP cells was incubated with Mito-Sox Red (5 μM) for 30 min at 37 ℃, then washed by PBS for three times and observed by a fluorescence microscope (Olympus Inc., Tokyo, Japan)

### Mitochondrial membrane potential (ΔѰM)

ΔѰM was assessed using the mitochondrial-specific fluorescent probe JC-1 (5,5’,6,6’-124 tetrachloro-1,1’,3,3’-tetraethyl-benzimidazolylcarbocyanine iodide) (Beyotime Biotechnology, C2006). Brief, NP cells were incubated with JC-1(5 μM) for 30 min at 37 ℃ and washed by PBS for three times and observed by a fluorescence microscope (Olympus Inc., Tokyo, Japan). JC-1 forms multimers in NP cells shown high red fluorescence suggesting a normal ΔѰM, Loss of ΔѰM results in a lower red fluorescence and a high green fluorescence as the dye shifts from multimeric form to monomeric state.

### Mito-tracker red staining

Mito-tracker red CMXRos (Molecular Probes, Thermo Fisher Scientific Inc.) was used to stain mitochondria in live cells. As previously described^19^, NP cells were incubated with Mito-tracker probes at the concentration of 50 nM for 30 min at 37 ℃. Then, nuclei were stained with Hoechst 33258 dye for 10 min at 37 ℃. NP cells were washed by PBS for three times and imaged with a fluorescence microscope fluorescence microscope (Olympus Inc., Tokyo, Japan) and fluorescence intensity was quantified by using Image J software 2.1 (Bethesda, MD, USA).

### Puncture-induced rat intervertebral disc degeneration model

Forty-two adult male SD rats (200–250 g) used for this study were randomly obtained from Experimental Animal Institute of Wenzhou Medical University. The rats were housed in a controlled environment under standard conditions of temperature and humidity and an alternating 12-h light and dark cycle. The 42 male SD rats were randomly divided into three groups, the control group (14 males), IDD group (14 males), salidroside + IDD group.

The accepted puncture-induced IDD rat model was chose in this study^49^. And the surgery was performed as previous described^31^. Rats in the IDD, salidroside + IDD groups were anaesthetized by intraperitoneal injection of 2% (w/v) pentobarbital (40 mg/kg). The coccygeal intervertebral spaces Co5-6, Co8-9were selected for the study. After the rat tail skin is disinfected with iodophors, 26-G needles were used to puncture the whole layer of AF though the tail skin. The needles were kept in the disc for 1 min. After surgery, the rats in salidroside group were immediately intraperitoneal injected with Salidroside (25 mg/kg) every 2 days until they were sacrificed, other rats were injected with same amount of saline every 2 days. The rats were daily monitored to ensure their well-being, and all animals were allowed free unrestricted weight bearing and activity.

### X-ray image acquisition

After 4 or 8 weeks of puncture, X-ray image was performed on all animals. All rats were anaesthetized by an intraperitoneal injection of 10% pentobarbital (40 mg/kg). The rats were fixed in a prone position for X-ray image by X-ray irradiation system (Kubtec). The disc height index (DHI) was determined using a published method^49^.

### Magnetic resonance imaging (MRI)

After 4 or 8 weeks of puncture, magnetic resonance imaging (MRI) was performed on all animals. All rats were anaesthetized by an intraperitoneal injection of 10% pentobarbital (40 mg/kg). The rats were fixed in a prone position for MRI, and then the finger-specific coil MRI mode was used for rat tails as previously described^31^. Then, MRI was performed on all rats to evaluate the signal and structural changes in sagittal T2-weighted images using a 3.0T clinical magnet (Philips Intera Achieva 3.0 MR). T2-weighted sections in the sagittal plane were obtained with the following settings that was previously published: fast-spin echo sequence with time to repetition (TR) of 5400 ms and time to echo (TE) of 920 ms; 320 (h) 9256 (v) matrix; field of view 260; and 4 excitations. The section thickness was 2 mm with a 0-mm gap. The MRIs images were evaluated by three double-blinded orthopaedic researchers who classified the extent of IDD according to the Pfirrmann grade^50^

### Histology analysis

Rats were killed at 0, 2, 4, or 8 weeks after surgery, the rats were killed by an intraperitoneal over-dosage of 4% pentobarbital, and the punctured segment (Co5-6, Co8-9) and non-punctured tails were collected. The specimens were decalcified and fixed in formaldehyde, dehydrated, and embedded in paraffin. The tissues were cut into 5-μm sections. Slides of each disc were stained with Safranin O-fast green (S-O) and haematoxylin and eosin (H&E). The cellularity and morphology of NP and AF were examined by a separate group of experienced histology researchers in a blinded manner using a microscope (Olympus Inc., Tokyo, Japan) and evaluated using a grading scale. The histologic score was based on the histological appearance of the characteristics of the NP and AF.

### Statistical analysis

The results were presented as mean ± S.D. Statistical analyses were performed using SPSS statistical software program 20.0 (IBM, Armonk, NY, USA). Data were analyzed by one-way analysis of variance (ANOVA) followed by Tukey’s test for comparison between control and treatment groups. Non-parametric data (Pfirrmann grading) were analyzed by the Kruskal–Wallis H test. *P* < 0.05 was considered significant.
