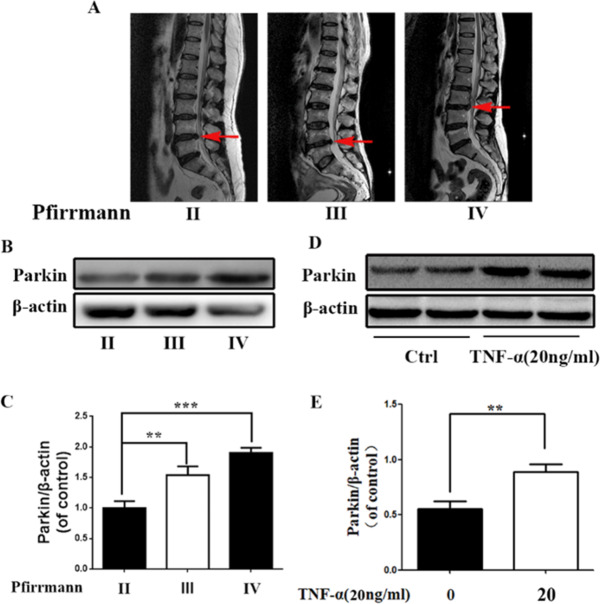# Correction: Parkin-mediated mitophagy as a potential therapeutic target for intervertebral disc degeneration

**DOI:** 10.1038/s41419-021-04183-9

**Published:** 2021-10-07

**Authors:** Zengjie Zhang, Tianzhen Xu, Jiaoxiang Chen, Zhenxuan Shao, Ke Wang, Yingchao Yan, Congcong Wu, Jialiang Lin, Haoli Wang, Weiyang Gao, Xiaolei Zhang, Xiangyang Wang

**Affiliations:** 1 Department of Orthopaedics, The Second Affiliated Hospital and Yuying Children’s Hospital of Wenzhou Medical University, West Xueyuan Road 109#, Wenzhou, 325027 Zhejiang Province China; 2 Zhejiang Provincial Key Laboratory of Orthopaedics, Wenzhou, Zhejiang Province China; 3 The Second School of Medicine, Wenzhou Medical University, Wenzhou, Zhejiang Province China; 4 The Third Affiliated Hospital and Ruian People’s Hospital of Wenzhou Medical University, Wansong Road 108#, Ruian, Zhejiang Province China; 5 Chinese Orthopaedic Regenerative Medicine Society, Ruian, China

Correction to: *Cell Death and Disease* 10.1038/s41419-018-1024-9, published online 24 September 2018

The original version of this article unfortunately contained a mistake. In the original version of this Article, the β-actin in Fig. 1B was shown incorrectly, this was due to an oversight at the typesetting stage. The authors have checked the original raw data and records of the experiments. These corrections do not affect the results and conclusions of this article. The authors would like to apologize for any inconvenience caused. The correct figure is as follows: